# Refractive Index Measurement of Glass with Arbitrary Shape Based on Brewster’s Law and a Focusing Probe Beam

**DOI:** 10.3390/s21072421

**Published:** 2021-04-01

**Authors:** Yao Hu, Jiahang Lv, Qun Hao

**Affiliations:** Beijing Key Lab. for Precision Optoelectronic Measurement Instrument and Technology, School of Optics and Photonics, Beijing Institute of Technology, Beijing 100081, China; huy08@bit.edu.cn (Y.H.); 3120205346@bit.edu.cn (J.L.)

**Keywords:** refractive index measurement, Brewster’s Law, focusing probe, image process, error analyses

## Abstract

The refractive index is one of the most important parameters of optical glasses and has a significant effect on optical properties. The measurement of optical glasses, especially for optical elements such as lenses, is urgently needed. However, several presented methods require the immersion of the sample in liquid and provide indirect measurements, while others require structural parameters as priori knowledge, which is complex and time-consuming. In this study, a Brewster-Law-based direct and simple measurement method for the refractive index of glasses with arbitrary shapes is proposed, and a laser beam is focused on the surface of the sample as a probe. The incident angle of the chief ray is close to the Brewster angle. The reflected light is collected by an array detector. The refractive index is calculated from the minimum intensity position obtained with image processing. Additionally, a symmetric measurement scheme is proposed to improve the accuracy. Using these methods, a prism and four spherical lens samples with different refractive indices or radii of curvature are tested and error analyses are carried out. Results indicate that the accuracy can reach 10^−4^.

## 1. Introduction

The refractive index, defined as the ratio of the velocity of light in a vacuum and the material, is one of the most important physical parameters of optical glass. With its considerable influence on the working characteristics of optical systems, high-accuracy measurement of this parameter is required. Since the early 1900s, various methods have been proposed [1], including prism coupling [2], critical angle [3], interferometry [4,5], and ellipsometry [6]. Some of them can even determine the refractive index and the structure of the sample simultaneously [7,8,9,10]. These methods are now widely used and can reach high accuracy of up to 10^−6^, but they are only available for prisms and parallel plates. Samples with only spherical or aspheric surfaces, such as lenses, cannot be tested. However, the measurement of the refractive index of a lens is equally important because of its possible changes during the manufacturing procedure, which may considerably affect the optical properties [11,12].

In the measurement of the refractive index of lenses, the liquid immersion method plays an important role. Smith [13] immersed a lens in a mixed liquid with varying refractive indices until it approximated that of the lens. Measuring the refractive index of the liquid can thus indirectly obtain that of the lens with an accuracy of 4.6 × 10^−4^. However, the mixed liquid used must be miscible, which is time-consuming for preparation. In addition, most of the miscible organic compounds are poisonous in nature. Other liquid immersion methods use non-miscible liquids. R. S. Kasana, et al. proposed a nondestructive technique using a Murty shearing interferometer [14]. The tested spherical lens is immersed in standard liquid with a certain refractive index inside a glass cell, from which the interferometric fringe is generated. The refractive index can be measured by analyzing the fringes with an accuracy of 10^−4^. Similarly, the refractive index of a lens can be measured by using computed tomography [15], Fabry–Perot interferometer [16], optical grating [17], acousto-optic grating [18], and Ronchi grating [19]. In these methods, the refractive index of the liquid does not need adjustments to fit that of the sample. However, the lens immersed shall be aligned to the optical axis, which is difficult in the liquid environment. Given that the refractive indices of the samples are obtained indirectly by measuring that of the liquid, the accuracy is limited by that of the liquid index measurement [20,21].

The non-immersive method is another important part of the refractive index measurement of lenses. The structural parameters of the tested lens—including focal length, thickness, and radius of curvature (ROC)—need to be measured primarily, and the refractive index is calculated by the geometrical formula. Vani K. Chhaniwal et al. calculated the refractive index of a thin biconvex lens by using the thin-lens formula and the structural parameters were measured by a Michelson interferometer [22] and digital holographic interferometry [23]. The accuracy was able to reach 10^−4^. However, the thin-lens formula is only available for biconvex lens with long focal lengths. Based on fiber point-diffraction longitudinal interferometry, Lingfeng Chen et al. [24] proposed a non-immersive method that can be used for any spherical single lens and reach an accuracy of 2.2 × 10^−4^. However, the system is based on an interferometry system that is sensitive to the environment. Weiqian Zhao et al. [25] proposed a multi-parameter comprehensive measurement method for spherical lenses using laser differential confocal interference, by which the structural parameters and the refractive index can be measured at the same time. The accuracy for the refractive index measurement can reach 2.2 × 10^−4^. However, this system is complex and expensive.

The Brewster method is a refractive index measurement method based on Brewster’s law [26,27,28], which can be expressed as the reflectivity of the P-polarized light approaching zero while it is incident in the Brewster angle, which is the inverse trigonometric value of the refractive index. In such a measurement system, a P-polarized parallel laser beam is incident on the sample and reflected to a photodiode to obtain the intensity. The minimum intensity position corresponding to the Brewster angle can be found by scanning the incident angle. The measurement accuracy of the refractive index depends on that of the angle [29,30]. The Brewster method enables simple and efficient measurement of the refractive index. However, the reflecting surface of the sample needs to be planar to provide a smooth reflective area with a size greater than the beam diameter. Thus, samples with only spherical or aspheric surfaces cannot be tested.

In this study, we propose a Brewster-Law-based direct and simple measurement method with a focusing probe beam for the refractive index of glasses with arbitrary shapes. A P-polarized laser beam is focused by a convergence lens on the surface of the sample as a focusing probe beam with a size of approximately 10 microns and then reflects to an array detector. The focusing probe beam is the incident light of the system, and the ray parallel to the optical axis of the convergence lens is defined as the chief ray. The incident angle of the chief ray is close to the Brewster angle. According to Brewster’s Law, a dark slit corresponding to the Brewster angle exists in the intensity distribution of the reflected light. Finally, image processing is used to calculate the Brewster angle and the refractive index by finding the minimum intensity position. The refractive index is measured by the reflection from an arbitrary surface of the sample; therefore, the structural parameters are not needed. Given that the probe requires only a tiny area for reflection, the method is available for samples with arbitrary shapes. Additionally, a symmetric measurement scheme is proposed to improve the accuracy. By using these methods, a prism and four spherical lens samples with different refractive indices or radii of curvature are tested and error analyses are carried out. Results indicate that the accuracy can reach 10^−4^.

This paper is organized as follows. The system structure and the measurement principle are presented in Section 2. In Section 3, the refractive indices of samples with different shapes are tested. The error analyses are presented in Section 4. Finally, conclusions are summarized in Section 5.

## 2. Measurement Principle

### 2.1. System Structure

**Figure 1:**
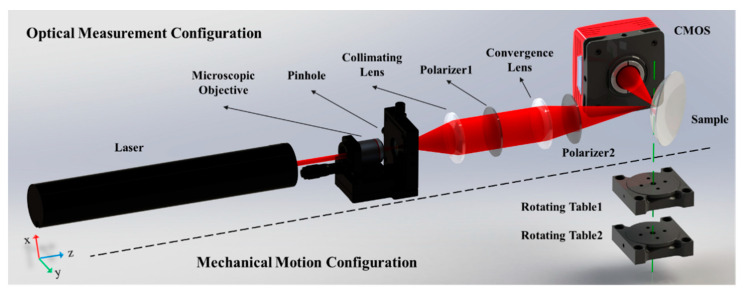
Layout of the refractive index measurement system using Brewster’s law and a focusing probe beam.

Figure 1 shows the system layout of the proposed refractive index measurement system, which is composed of optical measurement and mechanical motion configurations divided by the black dotted line. The optical measurement configuration is organized for the refractive index measurement, comprising two polarizers and a laser, microscope, collimating lens, convergence lens, sample, and a complementary metal oxide semiconductor (CMOS) array detector. The mechanical motion configuration consists of two coaxial rotating tables, marked as Rotating Table 1 and Rotating Table 2. The green dotted line is the common rotational axis of these tables, which passes through the surface vertex of the sample. Rotating Tables 1 and 2 take control of the sample and the CMOS, respectively.

**Figure 2:**
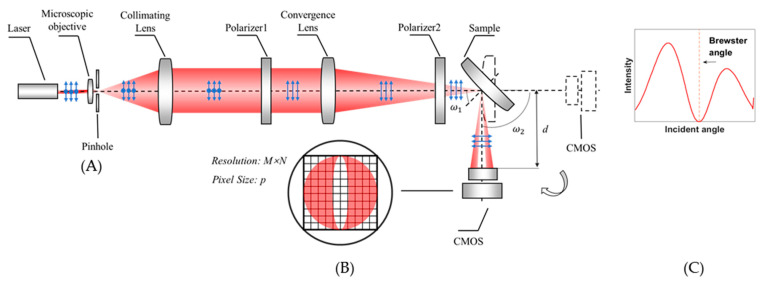
(**A**) Layout of the optical measurement configuration; (**B**) Intensity distribution of the reflected light; (**C**) Intensity curve of the reflected ray versus the incident angle.

Figure 2A shows the layout of the optical measurement configuration of the system. The thin laser beam passes through the microscopic objective and collimating lens to generate a broad collimated laser beam, which is used as a light source. The broad beam is focused by a convergence lens to the focal point as a focusing probe, which is coincident to the surface vertex of the sample. The incident angle of the chief ray of the probe is close to the Brewster angle. Polarizers 1 and 2, through which the light passes, are both in P polarization state, guaranteeing that the probe is a P-polarized beam. After the probe is reflected by the sample, the reflected light propagates to the CMOS detector placed at a distance *d* away from the sample. The resolution and the pixel size of the image plane are denoted as *M* × *N* and *p*, respectively.

Rotating the sample and the CMOS around the axis of the rotating tables can change the incident angle of the chief ray. The initial position of the sample and the CMOS is shown as a dotted line in Figure 2A. The rotation angle of the sample and the CMOS are marked as $\omega_{1}$ and $\omega_{2}$, respectively. Given that the incident angle is equal to the reflected angle, then $\omega_{2}{\;=\;180{}^{\circ}\;-\;2\omega }_{1}$. According to Snell’s Law, when the incident angle is close to the Brewster angle, the intensity distribution of the reflected light with a dark slit existing near the center can be illustrated as in Figure 2B. The center position of the distribution corresponds to the chief ray of the reflected light. Figure 2C shows the intensity curve versus the incident angle. The minimum position of the intensity curve corresponds to the Brewster angle.

**Figure 3:**
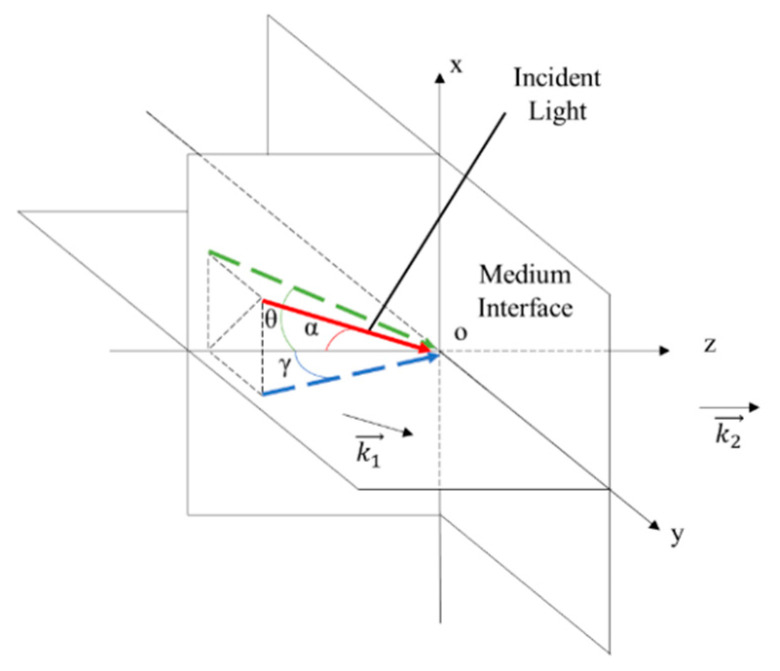
Geometric model of the incident focusing probe beam.

The reason for the dark silt formation is presented below. The intensity distribution of the reflected light is determined by the distribution of the incident light and the reflectivity, while that of the collimated laser beam is generally Gaussian and the reflectivity can be calculated by the incident angle. For the focusing probe beam, the incident angles vary at different positions, which follows the model in Figure 3.

In Figure 3, the xoy plane is the medium interface and the z axis is along the normal direction of the medium at the surface vertex. The red solid line is the incident light, while the green and blue dotted lines are the projection of the incident light on xoz and yoz plane, respectively. The incident angle is that between the incident light and the Z axis, which is marked as *α*. *θ* is the angle between the incident light and the xoz plane, and *γ* is the angle between the yoz plane. Vectors $\vec{k_{1}}$ and $\vec{k_{2}}$ are the normalized direction vectors parallel to the incident light and z axis, respectively, which can be expressed as

(1) $$\vec{k_{1}}\;=\;\rho \cdot \left(\sin\theta \cdot \cos\gamma ,\;\sin\gamma \cdot \cos\theta ,\;\cos\theta \cdot \cos\gamma \right),$$

(2) $$\vec{k_{2}}\;=\;\left(0,0,1\right),$$

where ρ is a normalized coefficient. The incident angle α can be calculated by

(3) $$\alpha \;=\;\arccos\left(\frac{\vec{k_{1}}\cdot \vec{k_{2}}}{\left|\vec{k_{1}}\left|\cdot \right|\vec{k_{2}}\right|}\right)\;=\;\arccos\left(\cos\theta \cdot \cos\gamma \right).$$

In Equation (3), the incident angle *α* changes with both *θ* and *γ*. Once either *θ* or *γ* is zero, *α* is equal to the other.

For the focusing probe beam in Figure 1, the focal point of the convergence lens corresponds to the coordinate origin of the model and the optical axis of the incident light corresponds to the Z axis. The chief ray of the incident light lies on the yoz plane, and thus the angle *γ* is zero and *θ* is equal to the incident angle *α*, which is close to the Brewster angle. Given that the incident angles of the rays at other positions of the beam distribute around the chief ray similarly to a cone, the values of *θ* and *γ* distribute around the Brewster angle and zero, respectively. The distribution range is determined by the focal length and diameter of the beam. Given that the reflected light is collected by the CMOS, *θ* and *γ* correspond to the row and column coordinates, respectively. After the *θ* and *γ* of each CMOS pixel are determined, the incident angle α can be calculated with Equation (3). According to Snell’s Law, the result that the reflected intensity distribution versus the incident angle consists of a dark slit with a minimum close to zero can be obtained.

According to the intensity distribution, an image processing method is used to find the minimum intensity position and the Brewster angle. Subsequently, the refractive index can be calculated. The detailed measurement of the Brewster angle and calculation of the refractive index are presented in Section 2.2.

### 2.2. Measurement of the Brewster Angle

**Figure 4:**
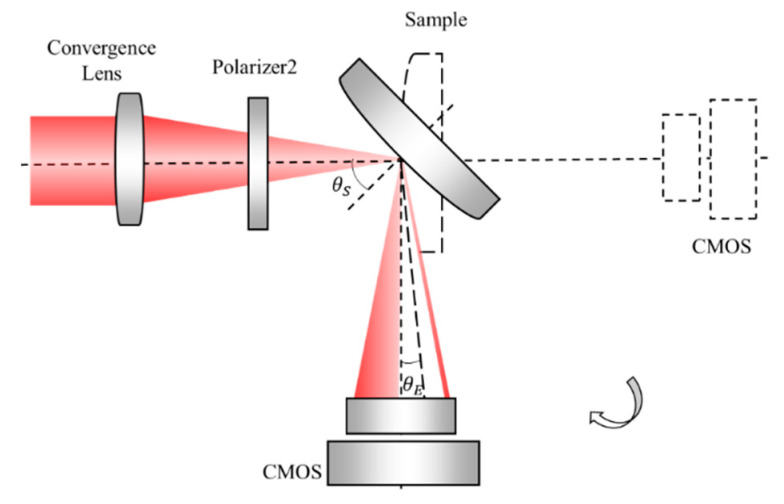
Incident model of the focusing probe beam.

The Brewster angle is determined using two steps. First, the sample and the CMOS are rotated such that a distinct dark slit appears in the image. In this case, the angle *θ* of the chief ray of the reflected light, marked as $\theta_{S}$ and equal to the rotating angle of the sample $\omega_{1}$, is measured by the readings of the rotating tables, as shown in Figure 4.

**Figure 5:**
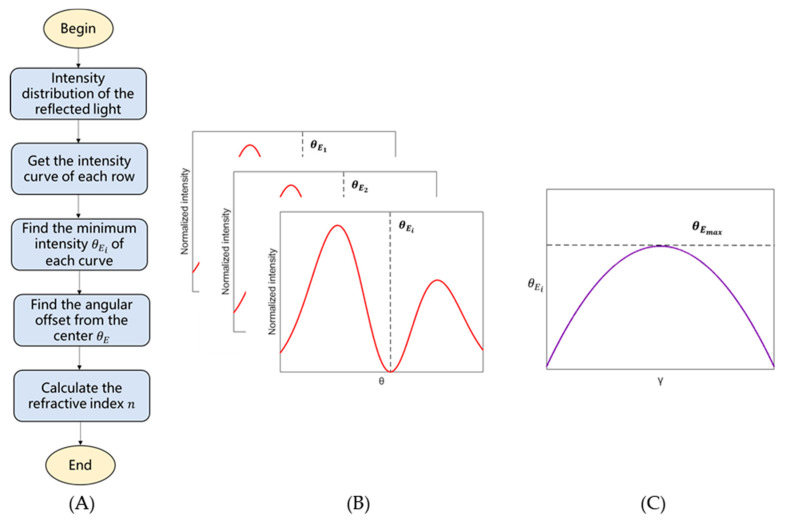
(**A**) Flow chart for calculating the refractive index; (**B**) Minimum intensity position $\theta_{E_{i}}$ in each row; (**C**) The maximum $\theta_{E_{\max}}$ corresponding to the angular offset from the center $\theta_{E}$.

Second, the angular offset $\theta_{E}$ between the chief ray corresponding to the horizontal center of the image and the Brewster-angle ray corresponding to the intensity minimum position is obtained by the image processing algorithm shown in Figure 5A. The first two steps draw the intensity distribution versus the column coordinate (representing *θ*) of each row, as shown in Figure 5B, and find the minimum intensity column coordinate in each row, marked as $\theta_{E_{i}}$, where *i* = 1, 2, … *M*. Generally, influenced by the angle *γ*, $\theta_{E_{i}}$ varies with the row coordinate *i*. The next step is finding the angular offset $\theta_{E}$. According to Equation (3), only when *γ* is zero is the incident angle *α* equal to *θ*. In other situations, *α* is greater than *θ*. If *γ* is farther from zero, *α* reaches the Brewster angle with a smaller *θ*. Thus, only in the row where *γ* is zero can *α* be equal to *θ* and $\theta_{E_{i}}$ reach a maximum. Figure 5C shows that the maximum value of $\theta_{E_{i}}$, denoted as $\theta_{E_{\max}}$, can be adopted to calculate the angular offset $\theta_{E}$ as

(4) $$\theta_{E}\;=\;\arctan[\frac{p\left(\theta_{E_{\max}}-\;\frac{N}{2}\right)}{d}].$$

Finally, after $\theta_{E}$ is found, the refractive index can be calculated as

(5) $$n\;=\;n_{0}\cdot \tan\left(\theta_{E}\;+\;\theta_{S}\right),$$

where *n* and $n_{0}$ are the refractive indices of the medium and of the air.

### 2.3. Symmetric Measurement Scheme

**Figure 6:**
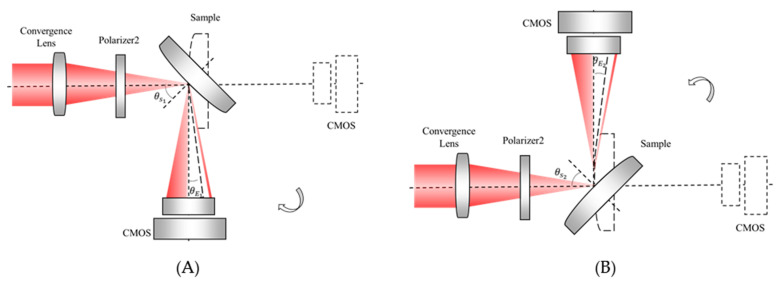
(**A**) Measurement of $\theta_{B}$ by clockwise rotation; (**B**) Measurement of $\theta_{B}$ by anticlockwise rotation.

According to the requirements of the system structure, the focal point of the convergence lens coincides with the surface vertex of the sample, and the common rotational axis of the two rotating tables passes through the surface vertex. These parameters are difficult to guarantee in real engineering. To reduce the alignment requirements of the system and guarantee high measurement accuracy, we introduce an additional measurement step, which is defined as the symmetric measurement scheme. The values of Brewster angle $\theta_{B}$ are obtained by rotating the sample and the CMOS clockwise and anticlockwise, as shown in Figure 6. The values are marked as $\theta_{B_{1}}$ and $\theta_{B_{2}}$, respectively. Finally, $\theta_{B}$ is determined as the average of the above two values as

(6) $$\;\theta_{B}=\frac{\theta_{B_{1}}+\theta_{B_{2}}}{2}=\frac{\theta_{E_{1}}+\theta_{E_{2}}+\theta_{S_{1}}+\theta_{S_{2}}}{2}.$$

This symmetric scheme can reduce the influence of several systematic errors. The verification is presented in Section 4.

## 3. Experiments and Results

### 3.1. Experiemental Setup

**Figure 7:**
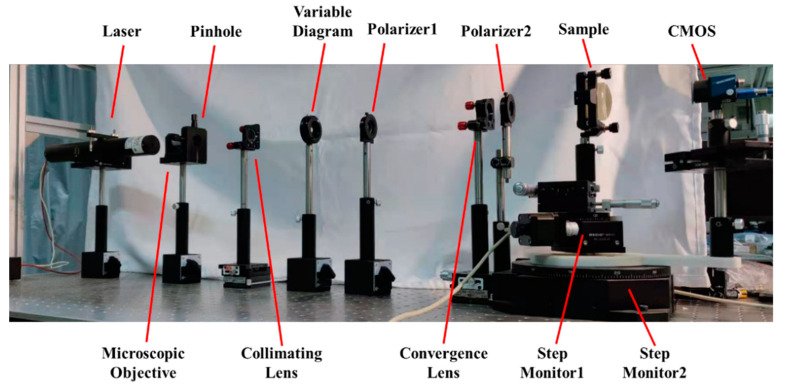
Experimental setup of the refractive index measurement system.

This section demonstrates the abovementioned principle with the refractive index measurement system, as shown in Figure 7. A He-Ne laser with a wavelength of 632.8 nm was used as the light source. Expanded by the microscopic objective, the pinhole, and the collimating lens, the diameter of the collimated laser beam was approximately 10 mm. The extinction ratio of the polarizers was 500:1. The focal length and the diameter of the convergence lens were 175 mm and 25.4 mm, respectively. The CMOS (Daheng Imaging, MER-302-56U3C) was placed at *d* = 150 mm from the focal point of the convergence lens, with *M* × *N* = 2048 × 1536 resolution and pixel size *p* = 3.45 μm. The sample and the CMOS rotations were realized by a pair of co-axis stepping motor rotating tables (BOCIC, MRS-101 and MRS-103). The stepping angle of each of the tables was around 1.3 × 10^−3^ degrees.

**Figure 8:**
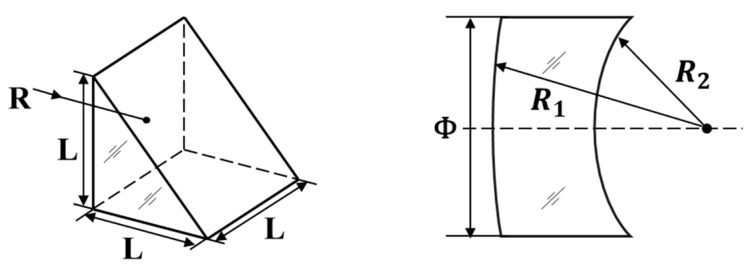
Structure of the tested samples.

Figure 8 shows the structure of the tested prism and spherical lens samples. Detailed information about the material, the reference value of the refractive index, and the structural parameters is listed in Table 1. The surface with ROC of $R_{1}$ was used for reflection in the experiment for the lens. The Rayleigh length of the focusing probe beam was around 160 μm, and the maximum sag variation in the target surface within the laser reflection area was around 7.5 μm, which was less than the Rayleigh length, proving that the reflection area could be treated as a point. The reference refractive index of the samples was settled from the glass database. The material H-K9L and N-BK7 were glasses with the same properties but produced by different companies, so their refractive indices were the same.

**Table 1:** Materials and structural parameters of the tested samples.

Serial Number	Shape	Material	Refractive Index (632.8 nm)	Diameter or Size Φ or *L*/mm	ROC $R_{1}/\mathrm{mm}$	ROC $R_{2}/\mathrm{mm}$
1	Prism	H-K9L	1.5151	25.4	∞	--
2	Lens	N-BK7	1.5151	25.4	1029.8	−1029.8
3	Lens	N-BK7	1.5151	25.4	515.1	∞
4	Lens	N-BK7	1.5151	25.4	386.3	∞
5	Lens	ZF7	1.7999	50.8	1074.58	41.54

We conducted two groups of experiments to verify the method and the system. In the first group, samples with the same refractive index but different shapes, i.e., samples 1, 2, 3, and 4, were tested to verify the validity for measuring the elements with arbitrary shapes. In the second group, samples with similar shapes (e.g., similar ROC of the surface for reflection) but different refractive indices, i.e., samples 2 and 5, were tested to estimate the accuracy and the consistency of the measurement of the different refractive indices.

### 3.2. Refractive Index Measurement of the Samples

**Figure 9:**
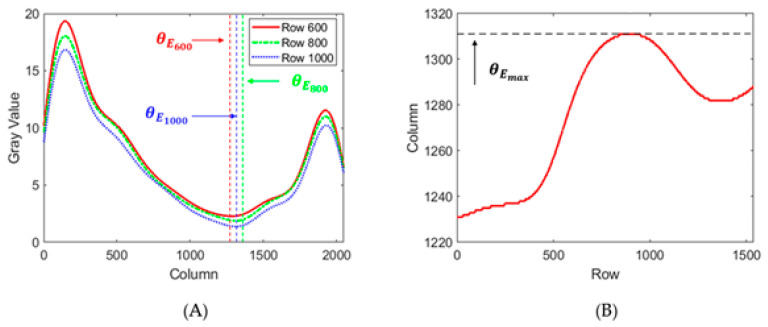
(**A**) Intensity curve in three typical rows in the measurement of sample 1; (**B**) Minimum intensity position curve in the measurement of sample 1.

Ten measurements were taken for each of the samples listed in Table 1, and each measurement took around five minutes because the mechanical rotation was not fully automatic. Figure 9 shows the data processing results of sample 1 as an example. Figure 9A is the intensity curve in three typical rows from the intensity distribution collected by the CMOS. The vertical axis is the grayscale value, and the horizontal axis is the column coordinate. The red, green, and blue curves correspond to the intensity curves in rows 600, 800, and 1000, respectively. In each curve, a minimum intensity position corresponding to $\theta_{E_{i}}$ can be found. The column coordinates of the curves are different, which accords with the principle in Section 2.1. Figure 9B shows the row and column coordinates of the minimum intensity position. The vertical axis is the column coordinate, and the horizontal axis is the row coordinate. Different values of $\theta_{E_{i}}$ make up the curve, and the maximum of the curve corresponds to $\theta_{E}$. The results for the other samples are similar. The detailed results and analyses are demonstrated in Section 3.2.1 and Section 3.2.2, respectively.

#### 3.2.1. Measurement of the Samples with Different Shapes

The results of the measurement of the samples made with H-K9L(N-BK7), including samples 1, 2, 3, and 4, are listed in Table 2.

**Table 2:** Results of the measurements of the samples made with H-K9L (N-BK7).

Sequence	Sample No. 1			Sample No. 2			Sample No. 3			Sample No. 4		
	RIC	RIAC	RIA	RIC	RIAC	RIA	RIC	RIAC	RIA	RIC	RIAC	RIA
1	1.5188	1.5105	1.5146	1.5186	1.5103	1.5145	1.5187	1.5104	1.5145	1.5187	1.5105	1.5146
2	1.5188	1.5104	1.5146	1.5188	1.5104	1.5145	1.5186	1.5107	1.5146	1.5185	1.5106	1.5145
3	1.5188	1.5104	1.5146	1.5188	1.5103	1.5145	1.5186	1.5104	1.5145	1.5188	1.5104	1.5146
4	1.5187	1.5105	1.5146	1.5189	1.5104	1.5146	1.5186	1.5103	1.5144	1.5184	1.5103	1.5144
5	1.5185	1.5104	1.5146	1.5188	1.5105	1.5147	1.5188	1.5104	1.5145	1.5188	1.5103	1.5145
6	1.5187	1.5104	1.5145	1.5188	1.5104	1.5146	1.5187	1.5105	1.5146	1.5186	1.5107	1.5147
7	1.5188	1.5104	1.5146	1.5187	1.5104	1.5146	1.5187	1.5106	1.5146	1.5185	1.5106	1.5146
8	1.5186	1.5103	1.5145	1.5187	1.5106	1.5147	1.5188	1.5104	1.5145	1.5186	1.5106	1.5146
9	1.5187	1.5104	1.5145	1.5188	1.5106	1.5147	1.5186	1.5105	1.5145	1.5188	1.5105	1.5146
10	1.5187	1.5104	1.5146	1.5188	1.5107	1.5147	1.5186	1.5106	1.5146	1.5188	1.5105	1.5146
Average	1.5187	1.5104	1.5146	1.5188	1.5105	1.5146	1.5187	1.5105	1.5145	1.5187	1.5105	1.5146
standard deviation (×10^−5^)	9.0	5.1	4.6	7.8	12.8	7.5	7.8	11.7	6.4	14.3	12.6	7.8

The refractive index measurement clockwise, the refractive index measurement anticlockwise, and the average refractive index are abbreviated as RIC, RIAC, and RIA, respectively, in the table.

For the measurements of sample 1, the reference value of the refractive index was 1.5151 at 632.8 nm. For the data collected clockwise, the average was 1.5187 and the standard deviation was 9.0 × 10^−5^. The average was approximately 3.6 × 10^−3^ greater than the reference, but the data had limited diversity. For the data collected anticlockwise, the average and the standard deviation were 1.5104 and 5.1 × 10^−5^, respectively. The average was 4.7 × 10^−3^ less than the reference. However, the diversity remained low. For the averaged result of clockwise and anticlockwise data, the average was 1.5146 and was only approximately 5.0 × 10^−4^ less than the reference. The standard deviation was 4.6 × 10^−5^.

For the measurement of samples 2, 3, and 4, the reference value of the refractive index was also 1.5151. The average refractive indices of the averaged result of clockwise and anticlockwise data were 1.5146, 1.5145, and 1.5146, respectively. Moreover, the standard deviations were 7.5 × 10^−5^, 6.4 × 10^−5^, and 7.8 × 10^−5^. The averages were approximately equal to that of sample 1, and the standard deviations were in the same order of magnitude. For the data collected clockwise and anticlockwise, similar conclusions could be drawn.

From the first group of experiments, we can conclude that for the samples with different shapes in the experiment, the results were approximately equal. The data collected clockwise were greater than the reference at 10^−3^ order, while for the anticlockwise data, the average was less than the reference at a similar order. The accuracy of the average data was around 5.0 × 10^−4^ to 6.0 × 10^−4^.

#### 3.2.2. Measurement of the Samples with Different Refractive Indices

The results of the measurement of the sample made with ZF7 (sample 5) are listed in Table 3.

For the measurements of sample 5, the reference value of the refractive index was 1.7999 at 632.8 nm. For the data collected clockwise, the average was 1.8055 and the standard deviation was 1.0 × 10^−4^. For the data collected anticlockwise, the average was 1.7953 and the standard deviation was 5.1 × 10^−5^. For the averaged result of clockwise and anticlockwise data, the average was 1.8004 and the standard deviation was 7.1 × 10^−5^. With and without the symmetry measurement, the accuracy can reach around 5 × 10^−3^ and 5 × 10^−4^, respectively.

Overall, the average of the data collected clockwise is greater than the reference at 10^−3^ order, while for the anticlockwise data, the average is less than the reference at a similar order. With and without using the symmetry measurement, the accuracy can reach 10^−3^ and 10^−4^, respectively. The second group of experiments indicated that for the samples with different materials, the results are relatively stable.

## 4. Error Analyses

### 4.1. Error Analyses of the System

According to Equation (5), the measurement error $\sigma_{n}$ of the refractive index of the sample is mainly caused by that of air $\sigma_{n_{0}}$, the angular measurement error of the incident angle at the center position $\sigma_{\theta_{S}}$, and the angular measurement error of the angular offsets $\sigma_{\theta_{E}}$.

#### 4.1.1. Refractive Index Measurement Error of Air${\;\sigma }_{n_{0}}$

The measurement error $\sigma_{nair}$ of the refractive index caused by the error of the refractive index of air can be obtained by differentiating Equation (5) with respect to $n_{0}$ as

(7) $$\sigma_{nair}\;=\left|\frac{\partial n}{\partial n_{0}}\cdot \sigma_{n_{0}}\right|,$$

where is $\sigma_{n_{0}}$ affected by the environment parameters, such as pressure, temperature, and humidity. Considering the parameters above, $\sigma_{nair}$ can be obtained by [31]

(8) $$\sigma_{nair}\;=\;{\left[{\left(2.68\times 10^{-9}\sigma_{Pa}\right)}^{2}+{\left(-9.27\times 10^{-7}\sigma_{K}\right)}^{2}+{\left(-1\times 10^{-8}\sigma_{H}\right)}^{2}\right]}^{1/2}\cdot \;\tan\left(\theta_{E}\;+\;\theta_{S}\right),$$

where $\sigma_{Pa}$, $\sigma_{K}$, and $\sigma_{H}$ are the errors of the pressure (pa), temperature (°C), and humidity (%, relative humidity).

#### 4.1.2. Angular Measurement Error $\sigma_{\theta_{S}}$

The measurement error $\sigma_{nS}$ of the refractive index caused by the angular measurement error $\sigma_{\theta_{S}}$ of incident angle $\theta_{S}$ can be obtained by differentiating Equation (5) with respect to $\sigma_{\theta_{S}}$ as

(9) $$\sigma_{nS}\;=\left|\frac{\partial n}{\partial \theta_{S}}\cdot \sigma_{\theta_{S}}\right|\;.$$

**Figure 10:**
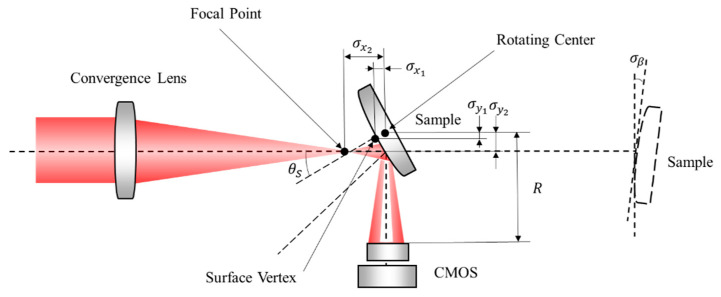
Error sources affecting the measurement error $\sigma_{\theta_{S}}$.

$\sigma_{\theta_{S}}$ is caused by various error sources, including the tilt angle $\sigma_{\beta }$, misalignment errors $\sigma_{x_{1}}$ and $\sigma_{y_{1}}$ of the surface vertex and the rotating center of the CMOS, and the misalignment errors $\sigma_{x_{2}}$ and $\sigma_{y_{2}}$ of the focal point and rotating center of the CMOS, as shown in Figure 10.

All of the above-mentioned are undetermined systematic errors, and thus $\sigma_{\theta_{S}}$ needs to be synthesized by the root square sum method. The angular measurement errors $\sigma_{\theta_{\beta }}$, $\sigma_{\theta_{x1}}$,$\;\sigma_{\theta_{x2}}$, $\sigma_{\theta_{y1}}$, $\sigma_{\theta_{y2}}$ caused by $\sigma_{\beta }$, $\sigma_{x_{1}}$, $\sigma_{x_{2}}$, $\sigma_{y_{1}}$, $\sigma_{y_{2}}$ can be respectively expressed as

(10) $$\sigma_{\theta_{\beta }}\;=\;-\sigma_{\beta }\;,\;$$

(11) $$\sigma_{\theta_{x1}}\;=\;\frac{\arcsin\left[\frac{1}{R}\cdot \sin\left(2\theta_{S}\right)\cdot \sigma_{x_{1}}\right]}{2},$$

(12) $$\sigma_{\theta_{x2}}\;=\;\frac{\arcsin\left[\frac{1}{R}\cdot \sin\left(2\theta_{S}\right)\cdot \left(\cos\theta_{S}-\sin\theta_{S}\cdot \tan\theta_{S}\right)\cdot \;\sigma_{x_{2}}\right]}{2}\;\;,$$

(13) $$\sigma_{\theta_{y1}}\;=\;\frac{\arcsin\left[\frac{1}{R}\cdot \sin\left(2\theta_{S}\right)\cdot \tan\theta_{S}\cdot \sigma_{y_{1}}\right]}{2}\;\;,$$

(14) $$\sigma_{\theta_{y2}}\;=\;\frac{\arcsin\left[\frac{1}{R}\cdot \left(\sin\left(2\theta_{S}\right)\cdot \tan\theta_{S}-\cos\left(2\theta_{S}\right)\right)\cdot \;\sigma_{y_{2}}\right]}{2}\;,$$

where *R* is the turning radius of the CMOS. The synthesis error of $\sigma_{\theta_{S}}$ is

(15) $$\sigma_{\theta_{S}}=\sqrt{\sigma_{\theta_{\beta }}^{2}+\sigma_{\theta_{x1}}^{2}+\sigma_{\theta_{x2}}^{2}+\sigma_{\theta_{y1}}^{2}+\sigma_{\theta_{y1}}^{2}}\;.$$

Finally, the measurement error $\sigma_{n_{S}}$ can be expressed as

(16) $$\sigma_{n_{S}}\;=\;\frac{\sqrt{\sigma_{\theta_{\beta }}^{2}+\sigma_{\theta_{x1}}^{2}+\sigma_{\theta_{x2}}^{2}+\sigma_{\theta_{y1}}^{2}+\sigma_{\theta_{y1}}^{2}}}{\cos^{2}\left(\theta_{E}+\theta_{S}\right)}\;\;.$$

#### 4.1.3. Angular Measurement Error $\sigma_{\theta_{E}}$

The measurement error $\sigma_{n_{E}}$ of the refractive index caused by the angular measurement error $\sigma_{\theta_{E}}$ of incident angle $\theta_{E}$ can be obtained by differentiating Equation (5) with respect to $\sigma_{\theta_{E}}$ as

(17) $$\sigma_{nE}\;=\left|\frac{\partial n}{\partial \theta_{E}}\cdot \sigma_{\theta_{E}}\right|\;.$$

In the measurement, $\;\sigma_{\theta_{E}}$ is mainly caused by the algorithm that is a random system error. Thus, $\sigma_{n_{E}}$ can be expressed as

(18) $$\sigma_{nS}\;=n_{0}\cdot \frac{\sigma_{\theta_{E}}}{\cos^{2}\left(\theta_{E}+\theta_{S}\right)}$$

#### 4.1.4. Synthesis Error

Considering the effect of the above-mentioned errors on the measurement result, the synthetic measurement error of the refractive index $\sigma_{n}$ is

(19) $$\sigma_{n}\;=\;\sqrt{\sigma_{nair}^{2}+\sigma_{nS}^{2}+\sigma_{nE}^{2}}.$$

### 4.2. Error Analyses of the Symmetric Measurement Scheme

In Section 2.3, we propose a symmetric measurement scheme to improve the system’s accuracy. The results show the effectiveness of the method. Here, the error analyses of the symmetric measurement are given to verify the method theoretically.

The error of the result obtained by Equation (6) consists of the angular measurement error $\sigma_{\theta_{S}}$ and $\sigma_{\theta_{E}}$. $\sigma_{\theta_{E}}$ is a random measurement error caused by the algorithm and does not change with or without the symmetric measurement. However, the error sources of $\sigma_{\theta_{S}}$, including $\sigma_{\beta }$, $\sigma_{x_{1}}$, $\sigma_{x_{2}}$, $\sigma_{y_{1}}$, $\sigma_{y_{2}}$, are all systematic errors that consist of positive or negative values. The error clockwise and anticlockwise are denoted as $\theta_{S_{1}}$ and $\theta_{S_{2}}$, respectively. For the quantitative evaluation of the errors, the angle approximation and equivalent infinitesimal can be used to simplify the equals as

(20) $$\theta_{S}\approx \theta_{S}-\sigma_{\beta }\approx \theta_{S}+\sigma_{\beta },$$

(21) η≈ arcsin(η),

where η is the contents of the square brackets in Equations (11)–(14).

Based on Equations (21) and (22), the expressions of the error coefficients of $\sigma_{\beta }$, $\sigma_{x_{1}}$, $\sigma_{x_{2}}$, $\sigma_{y_{1}}$, $\sigma_{y_{2}}$ for $\sigma_{\theta_{S1}}$, $\sigma_{\theta_{S2}}$, and$\;\sigma_{\theta_{S}}$ in Equations (10)–(14) can be simplified as listed in Table 4. With the symmetric measurement scheme, the errors from $\sigma_{\beta }$, $\sigma_{y_{1}}$, and $\sigma_{y_{2}}$ can be reduced, while those from $\sigma_{x_{1}}$ and $\sigma_{x_{2}}$ remain the same, verifying the method’s feasibility.

### 4.3. Error Budget of the Results

According to the experimental system presented in Section 3.1 and the results in Section 3.2, the error budget for the measurement result of the lens is carried out as follows.

First, the error estimation without the symmetric measurement scheme was conducted as proposed in Section 4.1. The measurement error $\sigma_{nair}$ caused by that of the refractive index of the air $\sigma_{nair}$ is calculated with Equation (8) as

(22) $$\sigma_{nair}\;\;\approx \;1.0685\times 10^{-5},$$

where the pressure is nearly a normal atmosphere, $\sigma_{Pa}$ ≈ 0; $\sigma_{K}$ = 6.4 °C, and $\sigma_{H}$ = 11.6%. The value of tan($\theta_{E}\;+\;\theta_{S}$) is equal to the reference refractive index.

According to the experimental system, Table 5 shows the estimated error limits of $\sigma_{\beta }$, $\sigma_{x_{1}}$, $\sigma_{x_{2}}$, $\sigma_{y_{1}}$, and $\sigma_{y_{2}}$. For the measurement of sample 5, $\theta_{S}$ is set at 1.0647 rad, and the turning radius R = 150 mm, equal to the distance between the CMOS and the rotating center. By the estimated error in Table 5 and Equations (10)–(16), the measurement error $\sigma_{nS}$ can be calculated as

(23) $$\sigma_{nS}\;\approx \;4.5109\times 10^{-3}$$

For the measurement of the lens sample, the calculation for $\theta_{Emax}$ shall be no greater than two pixels. Thus, the measurement error $\sigma_{\theta_{E}}$ can be estimated as $\sigma_{\theta_{E}}\;\approx \;0.0025{}^{\circ}$ according to Equation (4). $\sigma_{nE}$ can be obtained with Equation (18) as

(24) $$\sigma_{nE}\;\approx \;4.2396\times 0.0025\cdot \;\approx \;1.8499\times 10^{-4}$$

Finally, without the symmetric measurement scheme, the refractive index measurement error $\sigma_{n}$ is estimated with Equation (19) as

(25) $$\sigma_{n}\;\approx 4.6\times 10^{-3}.$$

The synthesis error matches the experiment results, which is approximately 5 × 10^−3^ for both clockwise and counterclockwise data.

Then, the error estimation with the symmetric measurement scheme as proposed in Section 4.2 is given. $\sigma_{nair}$ and $\sigma_{nE}$ are the same as in Equations (22) and (24). However, for $\sigma_{nS}$, the influence of $\sigma_{\beta }$, $\sigma_{y_{1}}$, and $\sigma_{y_{2}}$ can be reduced. $\sigma_{nS}$ can be obtained by

(26) $$\sigma_{nS}\;\;\approx 3.1417\times 10^{-4}\;.$$

With the symmetric measurement scheme, the refractive index measurement error $\sigma_{n}$ can be obtained by

(27) $$\sigma_{n}\;\approx 3.7\times 10^{-4},$$

which also matches the experimental accuracy of approximately 5 × 10^−4^. Finally, the accuracy of the refractive index measurement method with the symmetric measurement scheme can reach 10^−4^.

## 5. Conclusions

A refractive index measurement method using Brewster’s Law and a focusing probe beam is proposed in this study. A P-polarized laser beam is focused on the surface vertex of the sample as a probe with a size of approximately 10 microns and is then reflected to an array detector. According to Brewster’s Law, a dark slit corresponding to the Brewster angle exists in the intensity distribution of the reflected light. Image processing is used to calculate the Brewster angle and the refractive index. In addition, a symmetric measurement scheme is proposed to reduce the error introduced by alignment errors. A prism and four spherical lens samples with different refractive indices or radii of curvature were tested with the proposed method. The results indicated that the accuracy can reach 10^−4^.

Compared with the existing refractive index measurements for lenses, the proposed method has the following advantages:

1. Compared with the liquid immersion method, the refractive index can be tested directly in the air.
2. Compared with the current non-immersion method, the proposed method does not require the structural parameters of the tested samples. Thus, the method can be used regardless of the shape of the sample, and the measurement process is clear and simple.
3. The refractive index is tested by noninterference measurement, given that the method is robust to noise and environmental vibrations.

## Figures and Tables

**Table 3:** Results of the measurement of the samples made with ZF7.

Sequence	RIC	RIAC	RIA
1	1.8053	1.7953	1.8003
2	1.8054	1.7952	1.8003
3	1.8054	1.7953	1.8003
4	1.8055	1.7953	1.8004
5	1.8055	1.7952	1.8004
6	1.8055	1.7953	1.8004
7	1.8055	1.7953	1.8004
8	1.8057	1.7953	1.8005
9	1.8054	1.7954	1.8004
10	1.8055	1.7953	1.8004
Average	1.8055	1.7953	1.8004
Standard deviation	1.0 × 10^−4^	5.1 × 10^−5^	7.1 × 10^−5^

The refractive index measurement clockwise, the refractive index measurement anticlockwise, and the average refractive index are abbreviated as RIC, RIAC, and RIA, respectively, in the table.

**Table 4:** Error coefficients of the error sources in the expression of $\sigma_{\theta_{S1}}$, $\sigma_{\theta_{S2}},\;$and$\;\sigma_{\theta_{S}}$.

Error Sources	$\sigma_{\theta_{S1}}$	$\sigma_{\theta_{S2}}$	${\;\sigma }_{\theta_{S}}$
$\sigma_{\beta }$	−1	1	0
$\sigma_{x_{1}}$	$\frac{{\sin\theta }_{S}{\cdot \cos\theta }_{S}}{R}$	$\frac{{\sin\theta }_{S}{\cdot \cos\theta }_{S}}{R}$	$\frac{{\sin\theta }_{S}{\cdot \cos\theta }_{S}}{R}$
$\sigma_{x_{2}}$	$\frac{{\sin\theta \cdot (\cos}^{2}\theta_{S}{-\sin}^{2}\theta_{S})}{R}$	$\frac{{\sin\theta \cdot (\cos}^{2}\theta_{S}{-\sin}^{2}\theta_{S})}{R}$	$\frac{{\sin\theta \cdot (\cos}^{2}\theta_{S}{-\sin}^{2}\theta_{S})}{R}$
$\sigma_{y_{1}}$	$\frac{\sin^{2}\theta_{S}}{R}$	$-\frac{\sin^{2}\theta_{S}}{R}$	0
$\sigma_{y_{2}}$	$\frac{{3\sin}^{2}\theta_{S}{-\cos}^{2}\theta_{S}}{2R}$	$\frac{{-3\sin}^{2}\theta_{S}{+\cos}^{2}\theta_{S}}{2R}$	0

**Table 5:** Estimated error from the error sources of $\sigma_{\theta_{S}}$.

Error	Estimated Value	Units
$\sigma_{\beta }$	0.001	rad
$\sigma_{x_{1}}$	0.02	mm
$\sigma_{x_{2}}$	0.1	mm
$\sigma_{y_{1}}$	0.02	mm
$\sigma_{y_{2}}$	0.02	mm

## Data Availability

Not applicable.
